# Multiple mental representations in picture processing

**DOI:** 10.1007/s00426-021-01541-2

**Published:** 2021-06-10

**Authors:** Wolfgang Schnotz, Georg Hauck, Neil H. Schwartz

**Affiliations:** 1 Faculty of Psychology, University of Koblenz-Landau, Fortstr. 7, 76829 Landau, Germany; 2 Faculty of Communication and Environment, Rhein-Waal-University of Applied Sciences, Friedrich-Heinrich-Allee 25, 47475 Kamp-Lintfort, Germany; 3 Department of Psychology, California State University Chico, Chico, CA 95929 USA; 4 Present Address: Huxelrebenweg 118, 55129 Mainz, Germany

## Abstract

This article investigates whether goal-directed learning of pictures leads to multiple mental representations which are differently useful for different purposes. The paper further investigates the effects of prompts on picture processing. 136 undergraduate students were presented maps of a fictitious city. One half of the participants were instructed to learn their map as preparation to draw it from memory as precisely as possible (PrepDraw), which should stimulate the creation of an elaborated surface representation. The other half were instructed to learn the map as preparation for finding the shortest traffic connection from various locations to other locations (PrepConnect), which should stimulate the construction of a task-oriented deep-structure representation (mental model). Within both experimental groups, one-third of the participants received the map without prompts. Another third received the map with survey prompts (stimulating processing of what is where), and the final third received the map with connect prompts (stimulating processing of how train stations are connected). In the following test phase, participants received a recognition task, a recall task, and an inference task. For recognition and recall, two surface structure scores (extent, accuracy) and two deep structure scores (extent, accuracy) were calculated. The inference task served also to indicate deep structure accuracy. The PrepDraw group outperformed the PrepConnect group in terms of surface structure related variables, whereas the PrepConnect group outperformed the PrepDraw group in terms of deep structure-related variables. Map processing was not enhanced by prompts aligned with the instruction, but non-aligned prompts tended to interfere with learning.

**Supplementary Information:**

The online version contains supplementary material available at 10.1007/s00426-021-01541-2.

## Introduction

Abundant research has demonstrated that students learn better from text and pictures than from text alone (Ainsworth, 2014; Butcher, 2006; Carney & Levin, 2002; Johnson & Mayer, 2012; Mayer, 1997, 2009, 2014). This has been shown to apply to combinations of texts with very different kinds of pictures such as realistic pictures, diagrams, maps, and graphs. However, there is a marked inherent imbalance in this field: text processing has been studied now extensively for half a century. Most scholars share the view that readers who understand a text construct multiple mental representations in their mind: they are assumed to construct a mental representation of the text surface structure and a representation of the semantic deep structure. The latter, often referred to as the text base, consists of propositions representing the ideas expressed in the text. The propositional representation eventually serves as a data base for constructing a mental model of the text content. As these different kinds of mental representations are assumed to be differently useful for different purposes, learners put different emphasis on them depending on the kind of task they expect (Graesser et al., 1997; Kintsch, 1998; Kintsch & van Dijk, 1978; McNamara, 2007; van Dijk & Kintsch, 1983). By comparison, much less research has been devoted to picture processing (cf. Author, 1997; Cleveland, 1985; Glaser & Schwan, 2015; Glenberg & Langston, 1992). Do learners who understand a picture also construct multiple mental representations in their mind? Do they put different emphasis on the elaboration of these representations depending on their learning goals? Up to now, there is no clear answer to these questions (Boldini et al., 2007; Hockley, 2008).

## Theoretical framework

Previous research on learning from text and pictures resulted in theories assuming fundamentally different mental representations for text comprehension and for picture comprehension. One approach is represented by the Dual Coding Theory of Paivio (1986) which is referred to also by Kulhavy et al. (1985) in their Conjoint Processing Theory. The basic assumption is that text and pictures are processed in two cognitive sub-systems: a verbal system operating with so-called “logogens” and a pictorial system operating with “imagens”. Verbal information is processed and encoded only in the verbal system, whereas pictorial information is processed and encoded in the pictorial as well as the verbal system. Two codes are assumed to be better than only one code which would explain the superior memory for pictures compared to words. This view has become untenable, however, since it became obvious that text processing leads also to multiple codes (Kintsch, 1998; van Dijk & Kintsch, 1983).

Another approach is represented by the Cognitive Theory of Multimedia Learning (Mayer, 2009, 2014) which is partly derived from Dual Coding Theory. The theory proposes a working memory of limited capacity with an auditory-verbal channel for processing texts and a visual-pictorial channel for processing pictures. Processes of selection and organization result in a verbal mental model within the auditory-verbal channel and in a pictorial mental model within the visual-pictorial channel. If verbal and pictorial information are simultaneously in working memory, the two mental models can be integrated into an elaborated mental representation. Because two integrated mental models are assumed to be better than one model, it follows that learning from text and pictures is better than from text alone.

**Fig. 1:**
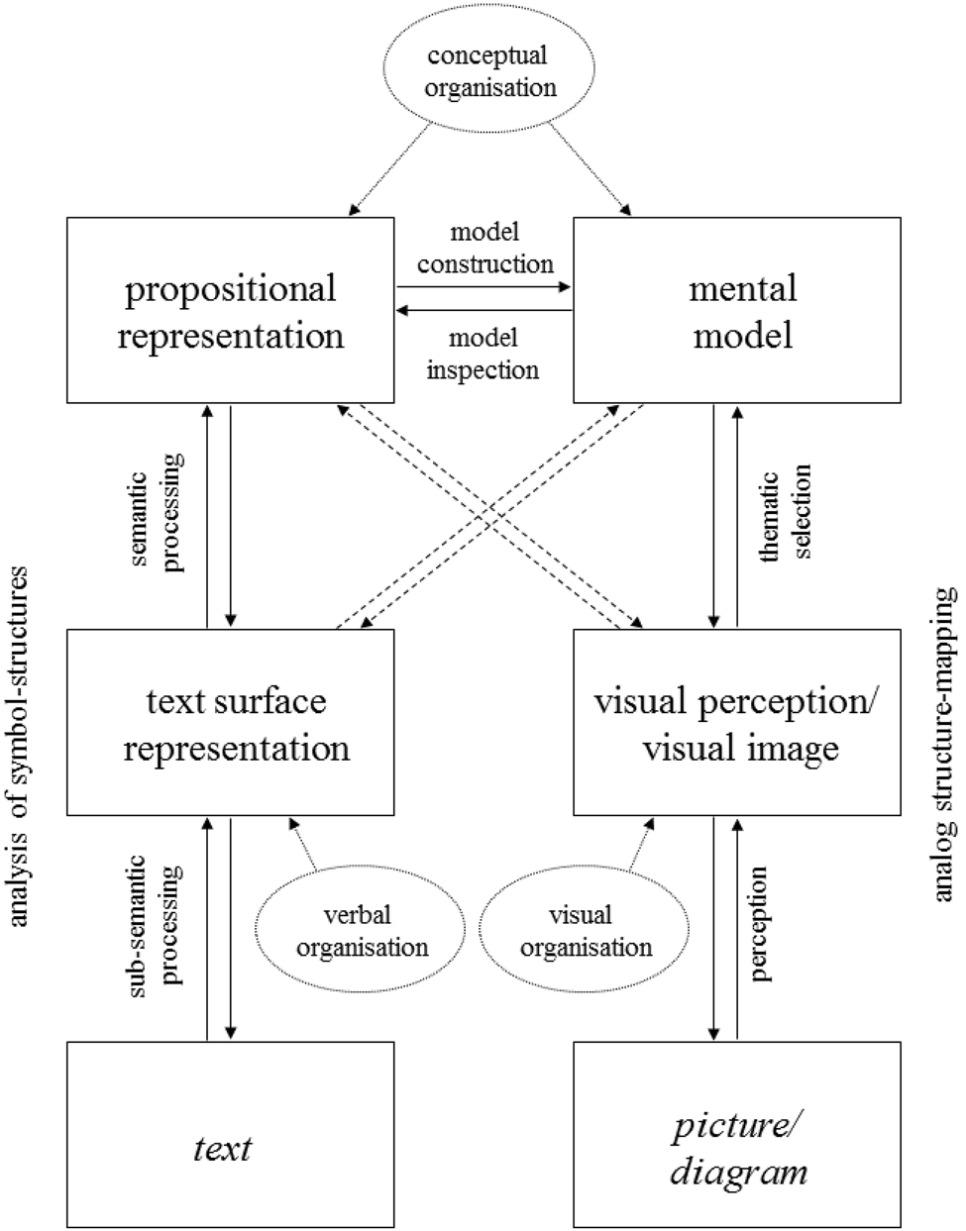
Integrated model of text and picture comprehension

The Integrative Model of Text-Picture Comprehension (ITPC-model) proposed by Schnotz and Bannert (2003, Schnotz 2014) is a theoretical framework for research on text and picture comprehension. The model assumes that both text comprehension and picture comprehension entail the formation of multiple mental representations. The model, which is shown in Fig. 1, incorporates the aforementioned standard view of text comprehension as well as supplementing assumptions about picture comprehension. Text and picture processing are assumed to take place in a working memory of limited capacity (Baddeley, 2003; Schroeder & Cenkci, 2019; Sweller et al., 2011, 2019) which includes a verbal (i.e., descriptive) channel and a pictorial (i.e., depictive) channel for the storage and processing of information.

The verbal channel includes the external text, the internal text surface representation, and the internal propositional representation of the semantic content of the text. The text surface representation and the propositional representation are internal descriptions, as they describe the represented object with the help of symbols (including relational symbols). Accordingly, information processing in this channel takes place by symbol processing. One can assume that verbal intelligence indicates to some extent the processing resources of this channel. The pictorial channel involves the external picture, the internal visual perception or image of the picture, and the internal mental model of the subject matter (Montgomery, 1988; Ohlsson, 1984a, 1984b). The visual image and the mental model are internal depictions, as their inherent structural characteristics are used for the purpose of representation. Accordingly, information processing in this channel takes place by structure mapping based on analogies (i.e., structural correspondences) between the depictive representations under the guidance of cognitive schemata which serve as scaffolds for mental model construction (Eitel et al., 2012; Gentner, 1989; Knauff & Johnson-Laird, 2002; Sims & Hegarty, 1997). One can assume that spatial intelligence indicates to some extent the processing resources of this channel.

The ITPC model assumes continuous interactions between the different representations. The interaction between a propositional representation and a mental model is assumed to take place via mental model construction processes and mental model inspection processes. These processes serve as “converters” between the descriptive channel and the depictive channel. In short, text and pictures are viewed as qualitatively different forms of representations processed in two different representation channels serving different functions in mental model construction (Schnotz et al., 2014; Shah et al., 2005).

### Multiple representations in picture comprehension

The ITPC model assumes different mental representations not only *between* text comprehension and picture comprehension, but also *within* both kinds of comprehension. As for picture comprehension, it assumes two kinds of quasi-spatial depictive representations: perceptual representations and mental models. A student processing a picture is assumed to create a perceptual representation through visual processing based on automated visual routines (Ullman, 1984). This kind of processing includes discrimination and identification of graphic entities as well as the visual organization of these entities according to the Gestalt laws (Wertheimer, 1938; Winn, 1990). The resulting visual perception is a surface representation of the picture. Because perception and imagery are based on the same cognitive mechanisms, the representation can be referred to as a visual image, when it is based on memory rather than external sensory data (cf. Kosslyn, 1994; Shepard, 1984). When students are required to solve specific tasks based on pictorial information, they will construct a task-oriented mental model through schema-driven mapping processes based on the picture’s perceptual representation. In this mapping process, graphic entities are mapped onto entities of the mental model (referred to as “tokens”), whereas spatial relations are mapped onto the semantic relations between the tokens within the mental model. Depending on the nature of the tokens, a mental model can be less or more abstract, whereby the level of abstraction might be strongly influenced by the intended usage of the model. The resulting mental model represents the depicted content by means of a structural or functional analogy (Johnson-Laird, 1983).

Referring to text processing terminology, where a distinction has been made between the surface structure and the deep structure^1^ of a text (the latter being equivalent to the propositional text base), we consider a perceptual representation of a picture as a representation of its surface structure and a mental model as a representation of its task-oriented deep structure. In short, the ITPC model considers picture comprehension as a process of analogical structure mapping between a system of visuo-spatial relations and a system of semantic relations (cf. Schnotz, 1993; Falkenhainer et al. 1989/90; Lowe et al., 2018; Rau et al., 2015).

Previous research has focused on various roles of pictures in the process of learning as, for example, the function of pictorial detail and its interaction with prior knowledge of learners (e.g., Dwyer, 1978), or the integrative function of maps in conjoint cognitive processing of text and pictures (e.g., Kulhavy et al., 1985). Other research aimed at the supporting function of pictures in mental model construction (e.g., Glenberg & Langston, 1992; Mayer, 2009) and at the influence of visualization formats on the structure of the emerging mental model (e.g., Schnotz & Bannert, 2003; Schnotz & Baadte, 2015). However, according to our knowledge, there is no empirical research yet on multiple mental representations within picture comprehension.

Perceptual surface representations and mental models are depictive representations, but they are qualitatively different. First, in terms of depth of processing, mental model construction (based on a perceptual representation) can be considered as a deeper level of picture processing than creating only a perceptual representation of the picture (Cermak & Craik, 1979; Rau, 2018). Secondly, perceptual representations are bound to a specific sensory modality, in picture processing usually the visual modality (Kosslyn, 1994). Mental models, on the contrary, are not sensory specific. A mental model of a spatial configuration, for example, can be constructed by visual or auditory or haptic perception. Because mental models are not bound to specific sensory modalities, they can be considered as more abstract than perceptual images, thereby allowing also different levels of abstraction. Third, mental models differ from visual images with regard to their information content. Whereas perceptual representations are less selective and more complete, mental models are, on the one hand, more selective and task-specific: only those parts of the visual configuration are included in the process of structure mapping which seem to be relevant for present or anticipated tasks. They can, therefore, be considered as the task-oriented deep structure of the picture. On the other hand, a mental model is elaborated through information from world knowledge and, thus, also contains information which is not included in the picture. For example, the mental model of a railway map would include information where the different trains run, where different trains take the same embankment, where trains stop, where passengers can change trains, etc., even when this so-called “self-evident” information is not explicitly stated in the map (Lobben, 2007).

### Goal-directed processing of pictures

Various researchers have demonstrated that text processing can be directed towards certain reading goals by instructing learners to prepare for specific tasks (Britt et al., 2018; McCrudden & Schraw, 2007; McCrudden et al., 2010; Pichert & Anderson, 1977; Rickards, 1979; Rickards & Denner, 1978; Rouet et al., 2017; Vidal-Abarca et al., 2010). The corresponding instruction triggers goal-directed processing which places special emphasis on task-relevant information.

We assume that picture processing is also a goal-directed strategic process. In view of the fact that different mental representations are differently useful for certain purposes, we hypothesize that individuals instructed to prepare for a specific task will try to construct task-appropriate mental representations. Accordingly, they will apply processing strategies directing their visual attention and cognitive processing on task-relevant information and they will put different emphasis on the creation of perceptual representations and on different mental models depending on the task at hand (cf. de Wit & Dickinson, 2009; Papenmeier et al., 2019; van der Laan et al., 2017).

When strategies are unfamiliar to learners (which could likely be the case with picture processing), it can be helpful to stimulate strategy usage with the help of prompts (Reisslein et al., 2006). Prompts are hints that suggest to engage in specific activities to put some strategy forward. The corresponding stimuli act as affordances. They can assume different forms such as directives or reminders. Prompts are usually aligned with the strategy the individual is expected to follow. Aligned prompts are meant to enhance the same kind or processing and to support the same task-oriented mental representation. The corresponding positive effect of aligned prompts can be called an “enhancement effect”. However, prompts can also be non-aligned with the strategy proposed by the instruction when the prompts are meant to further elaborate and complement the mental representation. Following an instruction and non-aligned prompts simultaneously, however, is a dual task which can impose a heavy cognitive load on working memory (Sweller et al., 2011). Non-aligned prompts can therefore interfere with the task-oriented processing induced by instruction, resulting in weaker task-oriented mental representations. The corresponding negative effect of non-aligned prompts can be called an “interference effect”.

Many kinds of pictures—among them especially maps—entail verbal labels that designate pictorial entities. These labels are not descriptions. They just help to identify the graphical entities and map them to the real-world entities they represent. According to the ITPC model, verbal labels are processed in the verbal channel, whereas the visuo-spatial information of pictures is processed in the pictorial channel of working memory. When verbal prompts are presented, these prompts have to be processed also in the verbal channel, where they draw from the processing capacity and reduce the available capacity for verbal labels (Castro-Alonso et al., 2019). Accordingly, the labels could therefore receive less attention which would impair learning of verbal components of picture (Author, 2007).

### Research questions and hypotheses

The research presented in this paper aimed at answering the following questions:

1. Do learners trying to understand a picture construct multiple mental representations in their mind? More specifically, do they create a perceptual representation of the picture’s surface structure as well as a mental model of the task-oriented deep structure and put different emphasis on the elaboration of these representations depending on the anticipating tasks or learning goals?
2. Can prompts affect strategies for goal directed picture processing? More specifically, do aligned prompts *enhance* task-oriented processing and do non-aligned prompts *interfere* with task-oriented processing of pictures? Do verbal prompts interfere with the learning of *verbal components* of pictures?

Regarding the first research question, we assumed that picture processing is focused on different mental representations depending on the goal of learning. If, for example, learners study a map and try to remember each and every detail as preparation to draw the map as precisely as possible from memory (abbreviated “PrepDraw”), they are expected to adopt a surface structure orientation focusing on the creation of a perceptual representation of the picture’s surface structure. If learners study the map as preparation to find the shortest connections for travelling from various locations to other locations with the public transport system (abbreviated “PrepConnect”), they are expected to adopt a deep structure orientation focusing on the construction of a mental model of the transport connections. Accordingly, PrepDraw learners were predicted to outperform PrepConnect learners with variables related to the surface structure, whereas PrepConnect learners were predicted to outperform PrepDraw learners with variables related to the deep structure.

Regarding the second research question, we assumed an enhancement effect of aligned prompts: PrepDraw learners with survey prompts were predicted to outperform PrepDraw learners without prompts with variables related to the surface structure. Conversely, PrepConnect learners with connect prompts were predicted to outperform PrepConnect learners without prompts with variables related to the deep structure. We also assumed an interference effect of non-aligned prompts: PrepDraw learners without prompts were predicted to outperform PrepDraw learners with connect prompts with variables related to the surface structure. Conversely, PrepConnect learners without prompts were predicted to outperform PrepConnect learners with survey prompts with variables related to the deep structure. Furthermore, we assumed an interference effect of prompts (aligned or non-aligned) on the learning of the picture’s verbal components: Participants who had received prompts were predicted to show lower recall of map labels compared to participants without prompts.

## Method

Permission for the study including ethical considerations was given by the faculty of psychology.

### Participants

137 undergraduate psychology students from a German university in their second year of studying aiming at a bachelor’s degree participated in the experiment. They had an average age of 23.5 years (SD = 3.8 years; range 19–44 years). 104 (76% of 137) were females; 33 (24% of 137) were males; 0 (0% of 137) were diverse. Participation was acknowledged as one of their study requirements. Students gave consent to participate in the study as contribution to improve learning material. A perquisite for participation was that students did not suffer from color-blindness.

### Experimental material

Within the large diversity of pictures, we decided to use maps as learning material. Maps are visual displays that represent a geographic space by reducing it to a pictorial space. They are particularly suited to incorporate multiple layers of information including two general affordances: *What is where? How to get from here to there?* (Lobben, 2007; Stanton et al., 2000; Tufte, 1990; Winn, 1991). In order to control for idiosyncratic peculiarities of a single map, we used four different schematic maps of a fictitious city. Each participant was assigned to one of these maps by systematic rotation.

**Fig. 2:**
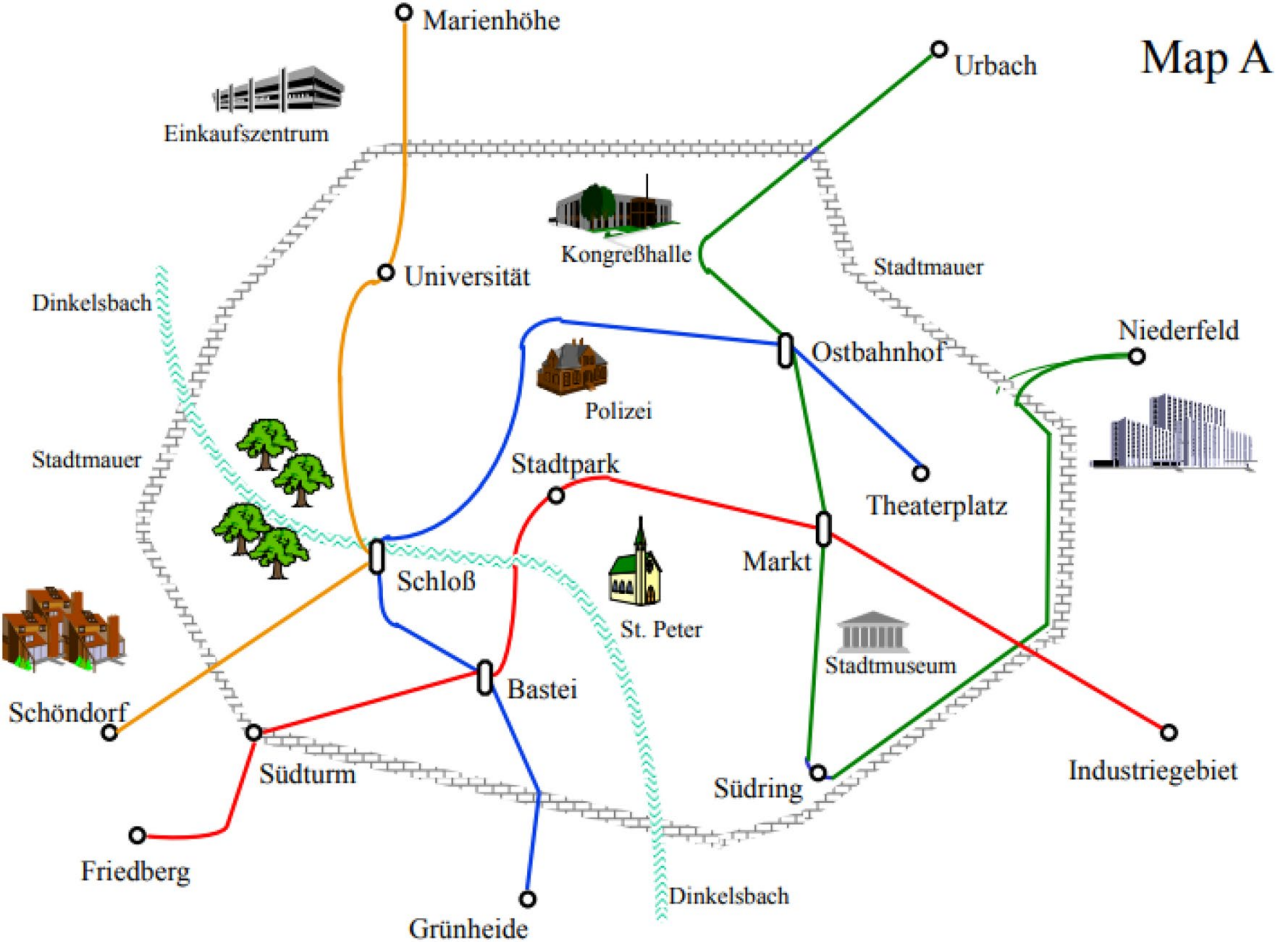
Example of a schematic map of a fictitious city used as learning material

**Fig. 3:**
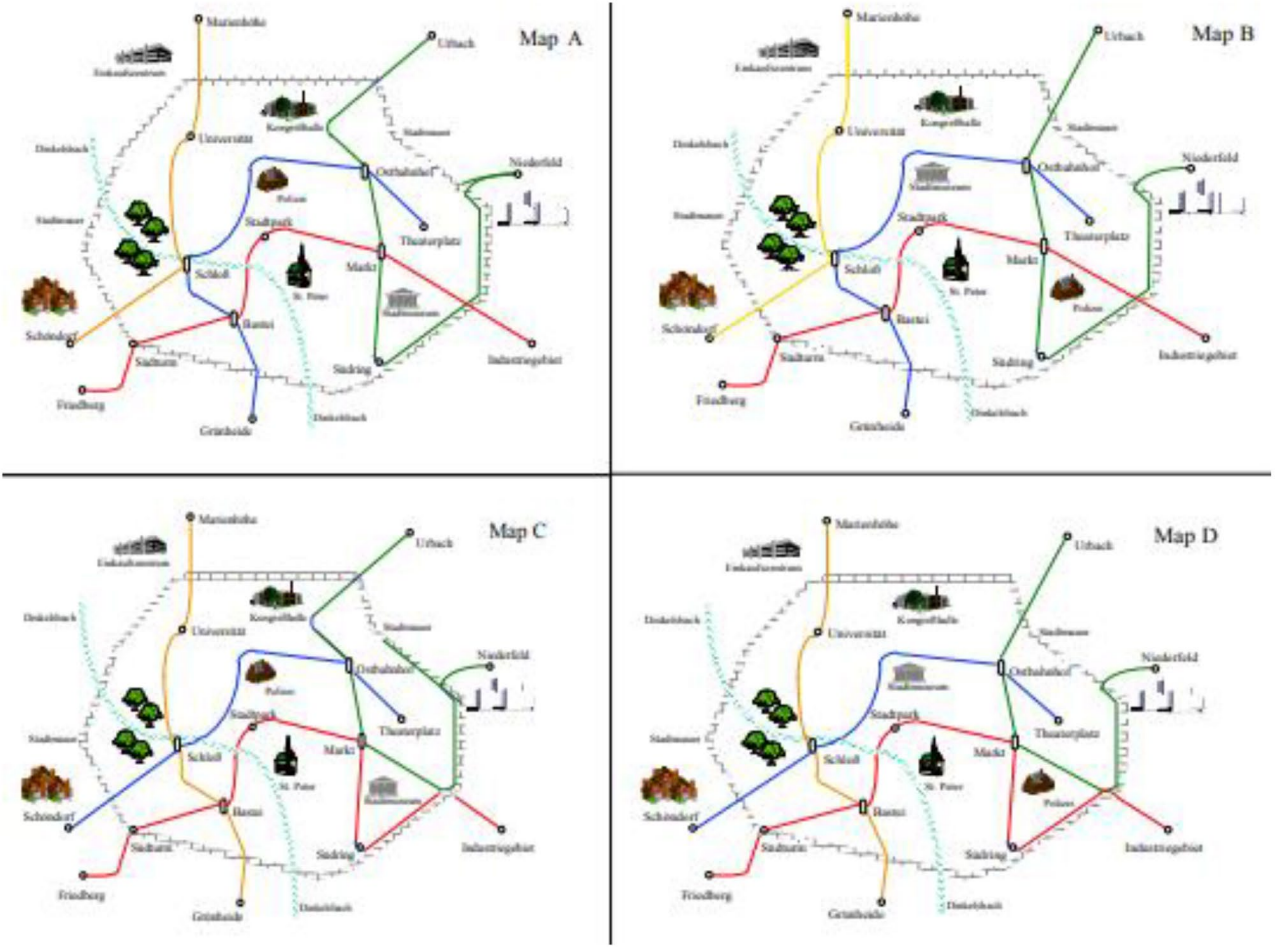
Overview of the four schematic maps of fictitious cities used as learning material

Figure 2 shows an example of these maps. The example will be referred to in the following as Map A. The maps showed city limits, a river, prominent landmarks through corresponding icons and the public transport network of the city. Different lines were indicated by different colors (yellow, red, blue, green). Each line included ordinary stops and transfer stations for changing trains. Figure 3 presents the whole set of the four maps (Map A to Map D).

The maps looked similar at a first glance but differed in two ways: Maps A and B had the same structure of transport connection, but were different in terms of surface features such as the location of buildings and the exact route of railroad lines. Maps C and D had another connection structure (different from A and B) but differed from each other in terms of surface features. So, A and C were similar in terms of surface features, but different in terms of connection structure. Likewise, B and D were similar in terms of surface features, but different in terms of their connection structure. If the connection structure is considered as the map’s deep structure, whereas the exact location of details makes up its surface structure, then A differed from B in terms of its surface structure, from C in terms of its deep structure, and from D in terms of surface and deep structure.

For each map, we created a survey prompt (aligned with PrepDraw processing) and a connect prompt (aligned with PrepConnect processing). The prompts took the form of short texts (length between117 and 157 words) indicating what *kind* of information should be extracted from the map. The prompts could not substitute the map, as they provided only guidelines for *how* to process the map and were far from being informationally equivalent. The survey prompts gave a short review emphasizing information about what is where. They were aligned with a PrepDraw instruction, but non-aligned with a PrepConnect instruction. The connect prompts emphasized information about how to get from one place to another place with the public transport connections indicated by the map. They were aligned with a PrepConnect instruction, but non-aligned with a PrepDraw instruction. Examples of a survey prompt and a connect prompt (referring to map A) are presented in Figures A1 and A2 in the Appendix.

### Design

Participants were randomly assigned to six experimental groups according to a 2 × 3 design with the between-factors *Instruction (2 levels)* and *Prompt (3 levels)*. Depending on their experimental treatment, the students were instructed to process the map with different goals in mind as preparation for different tasks. Participants of three subgroups were instructed to learn the map with a surface structure orientation: they were asked to learn each and every detail as preparation to draw the map from memory as precisely as possible. So, their main task was to learn what is where. Herein, this instruction will be called *Prepare for Drawing* (abbreviated “PrepDraw”). Participants of the other three subgroups were instructed to learn with a deep structure orientation: they were asked to learn as well as possible the shortest connections for travelling from various locations to other locations with the public transport system. This instruction will be called *Prepare for Connections* (abbreviated “PrepConnect”). So, their main task was to learn how to get from here to there. Two subgroups received their map without prompts, two subgroups with survey prompts and two subgroups with connect prompts.

### Procedure

Participants were tested before the experimental treatment in group sessions for their verbal and spatial intelligence with the Wilde-Test^2^ in order to control for comparability of experimental subgroups. The experiment took place 4–6 days later in single sessions which consisted of a learning phase and a test phase.

#### Learning phase

Participants were informed that they would be required to learn a map. Depending on their experimental treatment, they received their map with the instruction to prepare for drawing (PrepDraw) or with the instruction to learn the shortest connections of the public transport system (PrepConnect). They received their map either without prompt or with an instruction-aligned prompt or with a non-aligned prompt. Participants receiving a specific treatment were not informed about the other treatments. Participants had 4 min for learning the map. All materials were in German.

#### Test phase

After the learning phase, all participants received a recognition test, a recall test, and an inference test. Average time needed to finish all tests in the experiment was about 45 min. In order to control for interaction effects of sequencing, the order of the tests remained constant. Our assumption was that participants instructed to prepare for an exact reproduction of a map from memory will try to create an elaborated perceptual representation including all the details of the surface structure. Such a representation should be more complete rather than selective. Thus, it should manifest itself in better recognition of the original map compared to changes of the surface structure, a broader recall of map entities (including also details), and higher visuo-spatial accuracy of the reproduced map. By contrast, participants instructed to analyze public transport connections were assumed to construct a mental model which grasps the task-oriented deep structure of the visual display. Such a representation should be more selective. It should manifest itself in better recognition of the original map compared to changes of the deep structure, a more focused recall of map entities (excluding details), a more accurate recall of the task-oriented deep structure and higher performance in solving connection tasks. Accordingly, participants’ test performance was analyzed in terms of variables indicating the quality of the students’ mental surface structure representations and their deep structure representations. Scoring was done by research students who were not informed about the aim of the study. Measures were defined as follows.

### Measures

#### Recognition test

Participants were presented the maps A, B, C and D one by one in a fixed sequence which implies that the serial position of the original varied systematically with condition. Students had to judge each map as to whether it was the map they had previously seen (referred to as the “original”), or not. Furthermore, they had to indicate on a 5-point scale how confident they were about their judgment (ranging from “1 = very unconfident” to “5 = very confident”). They knew beforehand that one of the maps was the original.

*Surface structure and deep structure recognition scores.* To derive conclusions about mental representations from recognition data, we employed a paradigm suggested by Schmalhofer and Glavanov (1986), Fletcher and Chrysler (1990). Accordingly, the more the critical features of a mental representation have been elaborated by a learner, the better he or she will identify differences between a previously seen original and a distractor. Assume as an example that a student had been presented map A in the learning phase, which lets A be the original. Learners with a more elaborated perceptual representation should be better in discriminating surface differences (such as between maps A and B) than learners with a less elaborated representation: they should be less likely to falsely recognize B as the original and should also be more likely to detect that D is more different from the original A than C. Conversely, learners with a more elaborated mental model should be better in discriminating deep structure differences (such as between maps A and C) than learners with a less elaborated model: they should be less likely to falsely recognize C as the original and should be more likely to detect that D differs more from the original A than B (which in fact has the same deep structure as A).

On top of that, we assumed that more elaborated (i.e., better encoded) mental representations allow learners to be more confident in their judgments. Participants’ judgments (recognition or rejection) were therefore weighted with confidence. This resulted in a combined recognition-rejection scale (RR) ranging from “+ 5” (recognition, very confident) to “+ 1” (recognition, very unconfident)” for recognition and from “− 1” (rejection, very unconfident) to “− 5” (rejection, very confident) for rejection, which expressed the degree to which the participants accepted or rejected the map as the original. Based on this scale, recognition performances were defined via differences between the recognition-rejection scores (RR). Surface structure recognition was defined as [RR (A) − RR (B)] + [RR (C) − RR (D)]. Deep structure recognition was defined as [RR (A) − RR (C)] + [RR (B) − RR (D)]. Both scores were expected to vary between 0, which indicates no recognition and no confidence due to lack of or a weakly encoded mental representation, and 20, which indicates perfect recognition and high confidence due to a well encoded mental representation.^3^

#### Recall test

After the recognition test, participants performed a recall test. They received colored pencils (black, yellow, red, blue, green), an eraser and a sheet of A4-paper showing an empty map with only the river and the city limits. Participants were asked to complete the map from memory as well as possible.

#### Surface structure and deep structure recall scores

Participants’ drawings were analyzed in terms of which entities had been reproduced and labelled correctly. Because the mental model should include only entities of the connection structure, whereas the perceptual representation should include also the (connection-unrelated) details of the surface structure (i.e., landmarks such as police, museum etc.), the number of recalled details was used as an indicator for the elaboration of the perceptual representation. The pure number of reproduced entities was not indicative for the kind of underlying mental representation, because mental model as well as perceptual representation contribute to the recall of connection-related entities. The percentage of recalled entities related to the connection structure was used as an indicator of focused recall based on a mental model.

#### Surface structure recall accuracy scores

Visuo-spatial accuracy of the drawings was used as a further indicator of the quality of perceptual representations because these representations are assumed to preserve the visuo-spatial layout of a map’s entities. We measured in each participant’s drawing for each reproduced entity the Euclidean distance between its position in the original and its position in the drawing. Unit size was mm. Low average Euclidean distances were considered as indications of high surface structure accuracy.

#### Deep structure recall accuracy scores

Because the mental model is assumed to preserve the connection structure shown in the map, it should be a better basis for an accurate reproduction of the connecting structure than the perceptual representation which also includes other information. Accordingly, accuracy of the reproduced deep structure was considered as another indicator of the quality of the mental model. We determined in each participant’s drawing for each subway line the difference between the number of stops in the original and the number of stops in the drawing. Low differences summarized across subway lines were considered as indicating high deep structure accuracy.

#### Verbal recall accuracy scores

Labels on a map constitute verbal information which is expected also to be included when recalling the map. Thus, we determined for each participant’s drawing the number of missing or mixed-up labels compared to the original. Low numbers of missing or confused labels indicated high verbal accuracy.

#### Inference test

After the recall test, participants received the inference test which was made up of six connection tasks, each requiring to infer the shortest way to travel from a specific station X to another station Y by subway. Each of the six tasks was presented on a sheet of A4-paper with a kind of “empty map” showing only the river, the city limits, and the location of the stations that were to be connected by travelling in this specific task. Participants were required to specify which lines (color) have to be used, which stops are visited, and where a change of trains is needed in order to get from X to Y. For each item, participants had to write down their answers on the corresponding item sheet.

#### Inference accuracy scores

As mental models were assumed to allow inferences about how to travel from one place to another (Johnson-Laird, 1983), accuracy of inferences was used as indicator of the quality of the underlying mental model. The amounts of differences between the correct number of stops per item according to the original and the number of stops mentioned by the participant were added up across all connection items. Low difference sums were considered as indications of high inference accuracy.

## Results

In order to control for comparability of cognitive resources between different groups of participants, we compared them in terms of their verbal and spatial intelligence. Means and standard deviations of the groups to be compared are shown in Table 1 with the corresponding tests for comparability. As can be seen from the table, there was neither a significant difference in verbal intelligence nor a significant difference in spatial intelligence between the PrepDraw group and the PrepConnect group. The No-Prompt group differed neither significantly from the Aligned-Prompt group nor from the Non-Aligned Prompt group in terms of verbal intelligence. The same result was found for spatial intelligence. Thus, as the differences between conditions were small and far from significance, the relevant groups were well comparable in terms of their cognitive resources.

**Table 1:** Means and standard deviation of verbal and spatial intelligence and tests for comparability between treatment groups

PrepDraw	PrepConnect	No Prompt	Aligned Prompt	Non-aligned Prompt
Verbal intelligence				
*M* = 111.1	*M* = 112.7	*M* = 111.7	*M* = 111.3	*M* = 112.8
SD = 7.0	SD = 7.7	SD = 7.9	SD = 6.9	SD = 7.5
*n* = 69	*n* = 68	*n* = 49	*n* = 44	*n* = 44
Spatial intelligence				
*M* = 111.4	*M* = 112.7	*M* = 111.8	*M* = 111.5	*M* = 112.8
SD = 9.7	SD = 7.5	SD = 8.2	SD = 9.6	SD = 8.2
*n* = 69	*n* = 68	*n* = 49	*n* = 44	*n* = 44

PrepDraw vs. PrepConnect

Verbal intelligence: *t*(135) = 1.280, *p* = 0.20, *d* = 0.22

Spatial intelligence: *t*(135) = 0.928, *p* = 0.36, *d* = 0.16

No Prompt vs. Aligned Prompt

Verbal intelligence: *t*(91) = 0.301, *p* = 0.76, *d* = 0.06

Spatial intelligence: *t*(91) = 0.136, *p* = 0.89, *d* = 0.03

No Prompt vs. Non-aligned Prompt

Verbal intelligence: *t*(91) = − 0.670, *p* = 0.50, *d* = 0.14

Spatial intelligence: *t*(91) = − 0.670, *p* = 0.56, *d* = − 0.14)

The means and standard deviations of the abovementioned surface and deep structure variables in the PrepDraw group and the PrepConnect group under the different processing conditions are shown in Table 2. In order to test our assumptions regarding the first research question whether learners construct multiple mental representations, we performed a MANOVA of the surface structure variables (surface structure recognition, surface structure recall, and surface structure recall accuracy) as dependent variables as well as a MANOVA of the deep structure variables (deep structure recognition, deep structure focused recall %, deep structure recall accuracy, and inference accuracy) as dependent variables, both with factor *Instruction (PrepDraw/PrepConnect)* as independent variable. The results are presented in Table 3. All effects were significant and all of them were directed as expected in our hypotheses.

**Table 2:** Means and standard deviations of map learning variables by processing conditions

	PrepDraw			PrepConnect			Total		
	*M*	SD	*n*	*M*	SD	*n*	*M*	SD	*n*
Map only									
Surface structure recognition	7.24	8.10	25	3.79	5.55	24	5.55	7.12	49
Deep structure recognition	5.24	6.76	25	6.21	8.70	24	5.71	7.70	49
Surface structure recall	4.28	1.84	25	1.58	2.02	24	2.96	2.34	49
Deep structure focused recall [%]	67.1	16.6	25	87.0	15.2	24	76.9	18.7	49
Surface structure recall accuracy [mm]^a^	37.8	08.4	25	41.2	12.5	24	39.4	10.7	49
Deep structure recall accuracy^a^	8.92	5.92	25	5.08	4.21	24	7.04	5.46	49
Deep structure inference accuracy^a^	9.00	4.03	24	6.25	3.26	24	7.63	3.88	48
Verbal recall accuracy^a^	1.48	1.61	25	1.75	1.87	24	1.61	1.73	49
Map with survey prompt									
Surface structure recognition	5.00	6.47	22	3.73	7.48	22	4.36	6.94	44
Deep structure recognition	3.64	8.11	22	6.91	5.63	22	5.27	7.10	44
Surface structure recall	4.55	1.53	22	2.41	1.82	22	3.48	1.98	44
Deep structure focused recall [%]	62.5	15.1	22	78.2	16.2	22	70.3	17.4	44
Surface structure recall accuracy [mm]^a^	33.1	08.3	22	37.1	08.5	22	35.1	08.5	44
Deep structure recall accuracy^a^	7.55	3.97	22	7.59	5.74	22	7.57	4.88	44
Deep structure inference accuracy^a^	8.68	4.63	22	7.77	4.23	22	8.23	4.41	44
Verbal recall accuracy^a^	3.09	1.57	22	3.05	2.13	21	3.07	1.84	43
Map with connect prompt									
Surface structure recognition	4.00	6.44	22	1.55	6.70	22	2.77	6.61	44
Deep structure recognition	4.55	8.86	22	8.64	9.01	22	6.59	9.07	44
Surface structure recall	1.68	1.78	22	0.95	1.21	22	1.32	1.55	44
Deep structure focused recall [%]	85.8	12.9	22	91.8	9.39	22	88.8	11.6	44
Surface structure recall accuracy [mm]^a^	37.8	06.8	22	38.8	06.9	22	38.3	06.8	44
Deep structure recall accuracy^a^	7.77	4.45	22	5.09	1.66	22	6.43	3.59	44
Deep structure inference accuracy^a^	7.14	3.59	22	6.91	3.69	22	7.02	3.60	44
Verbal recall accuracy^a^	3.10	2.14	21	2.41	1.65	22	2.74	1.92	43
Total									
Surface structure recognition	5.49	7.13	69	3.04	6.58	68	4.28	6.94	137
Deep structure recognition	4.51	7.82	69	7.22	7.90	68	5.85	7.95	137
Surface structure recall	3.54	2.13	69	1.65	1.80	68	2.60	2.18	137
Deep structure focused recall [%]	71.6	17.8	68	85.7	14.9	68	78.6	17.8	137
Surface structure recall accuracy [mm]^a^	36.3	08.1	69	39.0	09.7	68	37.7	09.0	137
Deep structure recall accuracy^a^	8.12	4.87	69	5.90	4.32	68	7.01	4.72	137
Deep structure inference accuracy^a^	8.29	4.13	68	6.96	3.73	68	7.63	3.98	136
Verbal recall accuracy^a^	2.50	1.92	68	2.34	1.94	67	2.44	1.92	135

^a^Presented values specify deviation from correct. Accuracy is defined as the inverse of deviation from correct

**Table 3:** MANOVA results regarding the first research question: instructional effects on mental representations of surface structure versus deep structure

Hypotheses	Variables	Significance	Confirmed?
Surface structure representations			
PrepDraw > PrepConnect			
Univariate	Surface structure		
	Recognition Recall Recall accuracy	*F*(1,135) = 4.364, *p* = 0.02, *ƞ*^2^ = 0.03 *F*(1,135) = 31.330, *p* < 0.01, *ƞ*^2^ = 0.19 *F*(1,135) = 3.415, *p* = .03, *ƞ*^2^ = 0.03	Yes Yes Yes
Multivariate		*F*(3,133) = 10.480, *p* < 0.01, *ƞ*^2^ = 0.19	Yes
Deep structure representations			
PrepConnect > PrepDraw			
Univariate	Deep structure		
	Recognition Focused recall %	*F*(1,134) = 4.441, *p* = 0.02, *ƞ*^2^ = 0.03 *F*(1,134) = 24.817, *p* < 0.01, *ƞ*^2^ = 0.16	Yes Yes
	Recall accuracy	*F*(1,134) = 7.554, *p* = 0.01, *ƞ*^2^ = 0.05	Yes
	Inference accuracy	*F*(1,134) = 3.936, *p* = 0.02, *ƞ*^2^ = 0.03	Yes
Multivariate		*F*(4,131) = 6.647, *p* < 0.01, *ƞ*^2^ = 0.17	Yes

We also conducted a 2 × 3 MANOVA of the abovementioned surface structure variables and a 2 × 3 MANOVA of the abovementioned deep structure variables, both with factors *Instruction (PrepDraw/PrepConnect)* and *Prompt *[*No prompt/aligned prompt(non-aligned prompt)*] as independent variables. Despite the higher compartmentalization of variance, we found once again a significant effect of *Instruction* regarding the surface structure variables in favor of the PrepDraw group; *F*(3,129) = 13.091; *p* < 0.01; *ƞ*^2^ = 0.23. Conversely, we found a significant effect of *Instruction* regarding the deep structure variables in favor of the PrepConnect group; *F*(4,127) = 7.918; *p* < 0.01; *ƞ*^2^ = 0.20. As expected, the PrepDraw instruction resulted in a more elaborated (perceptual) surface structure representation than the PrepConnect instruction, whereas the PrepConnect instruction resulted in a more elaborated mental model as a deep structure representation than the PrepDraw instruction.

In order to test our assumptions regarding the second research question about enhancement and interference effects of prompts, we performed the corresponding MANOVAs with surface structure variables and deep structure variables as the dependent variables and the contrasts *Survey Prompt vs. No Prompt* and *Connect Prompt vs. No Prompt* as independent variables. The comparisons and their results are shown in Table 4. As can be seen from the table, none of the two comparisons testing for enhancement revealed a significant effect, whereas one of the two comparisons testing for interference found a significant difference as expected: connect prompts interfered significantly with the formation of a perceptual surface structure representation. On the contrary, there was no significant interference effect of survey prompts on deep structure representations.

**Table 4:** MANOVA results regarding the second research question: enhancement and interference effects of prompts

Enhancement	Variables	Significance	Confirmed?
PrepDraw:			
Survey Prompt > No prompt			
Univariate	Surface structure		
	Recognition Recall Recall accuracy	*F*(1,45) = 1.076, *p* = 0.31, *ƞ*^2^ = 0.02 *F*(1,45) < 1, *p* = 0.60, *ƞ*^2^ = 0.01 *F*(1,45) = 3.725, *p* = 0.06, *ƞ*^2^ = .08	No No No
Multivariate		*F*(3,43) = 1.937, *p* = 0.14, *ƞ*^2^ = 0.12	No
PrepConnect:			
Connect Prompt > No prompt			
Univariate	Deep structure		
	Recognition Focused recall %	*F*(1,44) < 1, *p* = 0.35, *ƞ*^2^ = .02 *F*(1,44) = 1.609, *p* = 0.21, *ƞ*^2^ = 0.04	No No
	Recall accuracy	*F*(1,44) < 1, *p* = 0.99, *ƞ*^2^ = .00	No
	Inference accuracy	*F*(1,44) < 1, *p* = 0.52, *ƞ*^2^ = 0.01	No
Multivariate		*F*(4,41) < 1, *p* = 0.58, *ƞ*^2^ = 0.07	No

Interference	Variables	Significance	
PrepDraw:			
Connect Prompt < No prompt			
Univariate	Surface structure		
	Recognition Recall Recall accuracy	*F*(1,45) = 2.259, *p* = 0.14, *ƞ*^2^ = 0.05 *F*(1,45) = 24.049, *p* < 0.01, *ƞ*^2^ = 0.35 *F*(1,45) = 0.0, *p* = 0.99, *ƞ*^2^ = 0.00	No Yes No
Multivariate		*F*(3,43) = 7.865, *p* < 0.01, *ƞ*^2^ = 0.35	Yes
PrepConnect:			
Survey Prompt < No prompt			
Univariate	Deep structure		
	Recognition Focused recall %	*F*(1,44) < 1, *p* = 0.75, *ƞ*^2^ = 0.00 *F*(1,44) = 3.679, *p* = 0.06, *ƞ*^2^ = 0.08	No No
	Recall accuracy	*F*(1,44) = 2.889, *p* = 0.10, *ƞ*^2^ = 0.06	No
	Inference accuracy	*F*(1,44) = 1.888, *p* = 0.18, *ƞ*^2^ = 0.04	No
Multivariate		*F*(4,41) = 3.120, *p* = 0.28, *ƞ*^2^ = 0.11	No

In view of the low number of cases within the single comparisons, these findings should be interpreted only tentatively and with great care. The lack of an enhancement effect seems to suggest that, on the one hand, picture processing received in this study sufficient direction by the instruction. In other words, it did not need further support by prompts. On the other hand, picture processing seems to be relatively vulnerable to stimuli not well aligned with the goal of processing. However, interference might not necessarily occur when prompts are not aligned with the instruction. In the present study, connect prompts interfered with the surface structure representation, but survey prompts did not interfere with the deep structure representation. The conditions of such specific interference effects will need further research.

Finally, in order to investigate whether verbal prompts would impair verbal map learning, we compared accuracy of verbal recall after learning without verbal prompts to recall after learning with verbal prompts (aligned as well as non-aligned). The average number of missing or confused labels when the map was presented without prompt was 1.6 (SD = 1.7), whereas it was 2.9 (SD = 1.9) when presented with prompts. The difference was significant; (*t*(133) = 3.964; *p* < 0.01; *d* = 0.72). Thus, accuracy of verbal recall was higher without verbal prompts which indicates that the verbal prompts can impair verbal map learning. Obviously, the prompts drew on the same cognitive capacity as the processing of the map labels. Thus, learners could likely concentrate less on the map labels, and the associations between entities and the corresponding labels became weaker. As a result, labels were more frequently missing or confused during recall.

All in all, we found little evidence that map learning would be positively influenced by aligned prompts, but an indication that non-aligned stimuli can easily interfere with picture processing.

## Discussion

The present study was based on the assumption that picture processing is a goal directed strategic process. We hypothesized that individuals instructed to prepare for a specific task will direct their attention and processing on task-relevant information and try to construct task-appropriate mental representations. The Integrative Model of Text-Picture Comprehension (ITPC model) was used as a theoretical framework for the study. According to this model, pictorial (visuo-spatial) processing takes place in the pictorial channel, whereas verbal processing (e.g., of verbal labels) takes place in the verbal channel of a working memory with limited capacity. The model suggests that learning from pictures includes the formation of a perceptual representation of the picture’s surface structure and, if needed, the construction of a more abstract task-oriented mental model of the depicted content. So, our research questions were: do learners create a perceptual representation of the picture’s surface structure as well as a mental model of the task-oriented deep structure? Do learners put different emphasis on the elaboration of these representations depending on the anticipating tasks or learning goals?

Our experiment confirmed the hypothesis that learners who try to understand a picture do construct multiple mental representations and put different emphasis on the elaboration of these representations depending on the anticipating tasks. Participants who were instructed to remember each and every detail (Prepare for Drawing) were expected to create a more elaborated (perceptual) surface structure representation and to outperform other students in surface-structure related measures of recognition and recall. Participants instructed to learn connections (Prepare for Connections), were expected to create a more elaborated mental model of the task-related deep structure and to outperform other students in deep structure-related measures of recognition, recall, and inferences. All predictions were confirmed by the data for all the indicator variables.

It should be noted that participants had to solve tasks they did not expect. Participants in the PrepDraw group had expected the drawing test, but had not expected the recognition test and had not expected the inference test. Participants in the PrepConnect group had expected the inference test, but had not expected the recognition test and had not expected the drawing test. Nevertheless, all contrasts were in line with our hypothesis as expected. Thus, the results cannot be explained simply by transfer-appropriate processing according to anticipated tasks. The consistent pattern of results no matter what the learners had expected can be considered as a hint that the performance differences were indeed due to different mental representations.

The notion that learning from pictures implies mappings of perceptual surface structures and semantic deep structures is in line with research of Knauff and Johnson-Laird (2002) who found that mental models differ from visual images and that different brain areas are involved in creating visual images and spatially organized mental models (Knauff et al., 2002, 2003). There is also evidence for a distinction between visual and spatial components in processing of verbal and pictorial information found by Gyselink et al. ().

When processing of pictures results in a representation of the surface structure and a representation of the (task-defined) deep structure, this could be considered as a commonality between picture processing and text processing, where a distinction is made between a mental text surface representation and a semantic deep structure representation, the so-called text base. Depending on the kind of expected tasks, these representations can also receive different emphasis during processing (Kintsch, 1998; van Dijk & Kintsch, 1983). However, the mental representations of text processing and those of picture processing seem to differ fundamentally in terms of forgetting rates. Text readers such as actors or singers can admittedly concentrate intentionally on the exact phrasing and store a verbatim representation of the text surface in long-term memory. Most frequently, however, readers deal with expository text and their aim is constructing a mental model of what the text is about. They seem to use the surface structure representation just as a means to extract the semantic content and encode it in the propositional text base. Accordingly, research on text comprehension and text learning has yet again found relatively high forgetting rates of text surface structure representations and much lower rates of forgetting of mental models (Graesser et al., 1997; Sachs, 1967). In picture comprehension, on the contrary, the perceptual surface representation seems to have a considerably longer duration (as compared to text processing) as indicated by learners’ drawn renditions after relatively short presentation and long retention times (e.g., Kulhavy et al., 1985). Similarly, in the present study, learners were after 15 or 20 min still able to deal well with surface-structure related tasks. These differences between mental representations need further investigation.

The findings regarding the effects of additional prompts on task-oriented processing were less clear cut. We found no indications of an enhancement effect of task-aligned prompts on picture processing. Learners had obviously adopted a certain strategy for picture processing based on the corresponding instruction and did not need further support by prompts. Picture processing seems to be relatively autonomous, as it can be sufficiently directed by a certain goal and does not need further support. The situation might be different, however, when an individual has to deal simultaneously with multiple tasks that make it hard to maintain a particular processing goal in mind. In this case, task-aligned prompts might very well have an important enhancement effect. This issue needs further research too.

Regarding possible interference effects of non-aligned prompts on picture processing, findings were more distinct. While goal-directed picture processing seems to be on the one hand relatively autonomous, it can on the other hand be vulnerable to stimuli not well aligned with the goal of processing. Connect prompts interfered with surface structure representations, while no interference of survey prompts was found with deep structure representations. The different results can possibly be explained by a different fragility of the corresponding picture processing strategies. As for the PrepDraw instruction, exact memorization of a picture is a relatively unusual task which means that participants had probably little pretraining. Furthermore, the to-be-learned content included a rich set of entities. Encoding their exact spatial locations might have been another challenge. Thus, usage of a surface-structure oriented strategy might be more fragile and vulnerable to external interference. As for the PrepConnect instruction, on the contrary, analysis of connections is a more straightforward task. Participants might have had more pretraining from looking at local traffic maps. Furthermore, the to-be-learned content included less entities: only lines, stops, and interchange stations. The relations were only simple bidirectional connections. Thus, usage of a deep-structure oriented strategy might be more straightforward, more stable and less vulnerable to external interference. This could explain why there was interference with surface structure representations but not with deep structure representations. This difference needs also further investigation.

Participants who had received verbal prompts had also significantly more missing or confused labels in their drawings than participants who had received no verbal prompts. All in all, we found little evidence that learning of the presented maps would be positively influenced by aligned prompts. However, we did find indications that non-aligned prompts can easily interfere with pictorial and verbal map learning. A closer analysis of this interference might yield also insights into effects usually associated with split-attention in multimedia learning.

The present study has various limitations which narrow possible generalizations. First, only one kind of pictures were used, namely maps associated with specific affordances corresponding to the questions *What is where?* and *How to get from here to there?* However, there are multiple other kinds of pictures with other affordances drawing the observer “automatically” into a specific direction. One can assume that comprehension of technical or scientific visualizations would similarly result in multiple pictorial representations, but it remains to be seen whether and to what extent this is the case. We suggest to conduct further studies with a broad spectrum of visualizations in order to check for the generalizability of specific findings.

A second limitation is that the different aspects of students’ performance (recognition, recall, inference) were evaluated in a fixed sequence. This raises the question whether there were transfer or interference effects between the dependent variables. One could argue, for example, that the time spent on the recognition task possibly influenced the outcome in the subsequent recall test. We cannot exclude this possibility, but we do not expect big differences resulting from such effects. The participants were shown four similar maps which were easy to be confused, and they did not know which of the four maps was the original. Thus, time on the recognition task was more likely time for interference (i.e., creating confusion) than time for additional learning. Interviews from a previous pilot study had shown that longer response times in the recognition task were due to participants’ uncertainty because of poor mental representations combined with carefulness and tentativeness. Thus, recognition response times were not correlated with recall performance. Nevertheless, future studies should vary the order of measuring the dependent variables.

A third limitation is that the study used only traditional off-line dependent variables to evaluate the quality of the learners’ mental representations: recognition, free recall, and inference tasks. These variables will certainly play also an important role in further investigations. However, as research on picture processing is still less developed in terms of methodology than research on text processing, further methods for data collection on picture processing and learning from pictures will be needed for further research. This could include on-line measures such as eye-movement studies in order to analyze the distribution of visual attention under different processing conditions. Eye-tracking techniques would also allow to estimate via comparison of accumulated fixation times the amount of effort invested by learners into processing the pictorial learning material under different instructional conditions. Data analysis could also include gestures and other forms of externalization of internal representations. Furthermore, methodology development should also include experimental paradigms in order to control specific parameters of picture processing.

A fourth limitation is that the learning time in this study was relatively short. This was partly due to methodological necessities: in order to investigate task-dependent differences between mental representations, we had to tune processing conditions in order to avoid floor effects as well as ceiling effects. Nevertheless, learning a map in four minutes is admittedly not a typical classroom task. Although learning from pictures consists frequently of multiple short time intervals resulting in multiple mental representations depending on the learning tasks at hand, studies of picture processing should also deal with more complex visual displays which require longer learning intervals. A fifth limitation is related to the relatively narrow scope of participants, who were psychology students. These students had no special training in reading maps. Although they might all have been familiar with reading local public transport maps, they probably did not have sophisticated strategies of map learning. This might also limit the generalizability of our findings. One could expect that other groups of learners such as architects or urban planners have due to their professional expertise more sophisticated cognitive schemata for analyzing such maps and would yield different results.

Currently, the findings of the present study seem to be relevant primarily for theoretical reasons. They support the view that picture processing is a goal directed strategic process which aims at constructing task-appropriate mental representations. Similar to text processing, processing of pictures seems to include the construction of multiple mental representations. These representations seem to include a perceptual representation of the picture’s surface structure as well as a mental model of the task-oriented deep structure. As these representations are differently useful for different purposes, learners put different emphasis on the elaboration of these representations depending on the anticipating tasks or learning goals.

Due to the limitations of the present study, the findings have of course to be examined by further investigations presenting other types of pictures, including other types of affordances, presenting other types of tasks, and using larger sample sizes (Pinker, 1990). These studies should also include other settings such as formal learning situations like schooling (e.g., lessons in geography) or informal learning situations such as museums. Furthermore, they should vary participants’ prior knowledge systematically. In addition, drawing might be a powerful tool to improve learning (Schmeck et al., 2014; Van Meter & Garner, 2005). Whereas drawing was only used in the present study as a diagnostic tool to reveal participants’ mental representations, future studies could present graphics in settings which allow also active drawing as a part of learning.

From a more general perspective, the present study has shown that learning from pictures is an instance of active sense making (Mayer, 2009; Wittrock, 1989), in which learners engage in constructing coherent mental representations from available information in order to meet certain requirements. We have to expect complex interactions between graphic formats, perceptual representations, mental models, and the learners’ cognitive schemata used to analyze pictorial material. These interactions will likely co-determine cognitive processing in learning from pictures. A deeper understanding of perceptual and cognitive processes underlying picture comprehension will hopefully improve our possibilities to develop guidelines for using different kinds of pictures in visual knowledge communication both from the side of designers and the side of learners.

## Supplementary Information

Below is the link to the electronic supplementary material.

Supplementary file1 (DOCX 15 kb)

Supplementary file2 (DOCX 15 kb)

## Footnotes

1 The idea of deep structures of sentences was introduced by Chomsky (1965) and initially considered as a syntactic concept. It was then re-interpreted by theorists such as Fillmore (1968) and Chafe (1970) as a semantic construct representing the meaning of sentences.

2 The Wilde-Test is a German standardized test for the measurement of cognitive skills of adults including verbal and spatial intelligence according to the standards of the German Psychological Society, based on the Modified Model of Primary Mental Abilities.

3 2*x*[(+ 5) − (− 5)] = 20.
