# Supplementary material for: Multiple mental representations in picture processing
Source: Psychol Res. 2021 Jun 10;86(3):903–18. doi: 10.1007/s00426-021-01541-2 (PMC8942916; doi:10.1007/s00426-021-01541-2)
Supplement: Supplementary file 1 — Supplementary file1 (DOCX 15 kb) [file 426_2021_1541_MOESM1_ESM.docx]

*Authors current themes of research*

Multimedia learning, text-picture integration, learning from animation, text comprehension, learning and problem solving in web-based environments, metaphorical graphics in instruction.

*Authors most relevant publications in the field of Psychology of Education*(ordered by recency)

Zhao, F., **Schnotz, W.,** Wagner, I., & Gaschler, R. (2020). Eyes on text and pictures – construction and adaptation of mental models. *Memory & Cognition, 48*(1), 69–82.

**Schnotz, W.,** & Wagner, I. (2018). Construction and Elaboration of Mental Models Through Strategic Conjoint Processing of Text and Pictures. *Journal of Educational Psychology, 110*(6), 850-863.

**Schnotz, W.,** Wagner, I., Ullrich, M., Horz., H., & McElvany, N. (2017). Development of students’ text-picture integration and reading competence across grades 5–7 in a three-tier secondary school system: A longitudinal study. *Contemporary Educational Psychology 51*, 152–169.

Wagner, I., & **Schnotz, W.** (2017). Learning from static and dynamic visualizations: What kind of questions should we ask? In R. Plötzner & R. Lowe (Eds.), *Learning from dynamic visualization* (pp. 69-91). Cham: Springer.

**Schnotz, W.,** & Baadte, C (2015). Surface and Deep Structures in Graphics Comprehension. *Memory & Cognition, 43*(4), 605-618.

Danielson, R. W., **Schwartz, N. H.,** & Lippmann, M. (2015). Metaphorical Graphics Aid Learning and Memory: The Role of Text-Graphic Correspondence in Learning. *Learning and Instruction, 39*, 194-205.

**Schnotz, W.** (2014). Integrated Model of Text and Picture Comprehension. In R.E. Mayer (Ed.), *Cambridge Handbook of Multimedia Learning (2nd Edition),* pp. 72-103. Cambridge: Cambridge University Press.

**Schnotz, W.,** Mengelkamp, C., Baadte, C., & **Hauck, G.** (2014). Focus of attention and choice of text modality in multimedia learning. *European Journal of Psychology of Education, 29*(3), 483-501.

**Schnotz, W.,** Ludewig, U., Ullrich, M., Horz, H., McElvany, N., & Baumert, J. (2014). Strategy shifts during learning from texts and pictures. *Journal of Educational Psychology. 106*(4), 974-989.

Zumbach, J. & **Schwartz, N.H.** (2014). Hyperaudio Learning for Non-Linear Auditory Knowledge Acquisition: A Comparison of Text Type, Presentation Format, and Modality. *Computers in Human Behavior 41*, 365-373.

Angeli, C., & **Schwartz, N.H.** (2014). Differences in Electronic Exchanges in Synchronous and Asynchronous Computer-Mediated Communication: The Effect of Culture as a Mediating Variable. *Interactive Learning Environments,* 1-22.

**Schwartz, N.H.,** Scott, B. M., & Holzberger, D. (2013). Metacognition: A Closed-Loop Model of Biased Competition–Evidence from Neuroscience, Cognition, and Instructional Research. In Azevedo & Aleven (Eds.) *International Handbook of Metacognition and Learning Technologies* (pp. 79-94). New York: Springer.

Hochpöchler, U., **Schnotz, W.,** Rasch, T., Ullrich, M., Horz, H., McElvany, N., Schroeder, S., & Baumert, J. (2013). Dynamics of Mental Model Construction from Text and Graphics. *European Journal of Psychology of Education, 28*(4), 1105-1126.

**Schnotz, W.** & Lowe, R.K. (2008). A unified view of learning from animated and static graphics. In R.K. Lowe & W. Schnotz (Eds.), *Learning with animation. Research implications for design* (pp. 304-356). New York: Cambridge University Press.

**Schnotz, W.** & Kürschner, C. (2007). A reconsideration of cognitive load theory. *Educational Psychology Review, 19*(4), 469-508.

Kürschner, C., Seufert, T., **Hauck, G., Schnotz, W.** & Eid, M. (2006). Konstruktion visuell-räumlicher Repräsentationen beim Hör- und Leseverstehen. *Zeitschrift für Psychologie, 214*(3), 117-132.

Kürschner, C., **Schnotz, W.,** Eid, M., & Hauck, G. (2005). Individuelle Modalitätspräferenzen beim Textverstehen: Präferenzen für auditive oder visuelle Sprachverarbeitung in unterschiedlichen Bevölkerungsgruppen. *Zeitschrift für Entwicklungspsychologie und Pädagogische Psychologie, 37*(1), 2-16.
