# Supplementary material for: Multiple mental representations in picture processing
Source: Psychol Res. 2021 Jun 10;86(3):903–18. doi: 10.1007/s00426-021-01541-2 (PMC8942916; doi:10.1007/s00426-021-01541-2)
Supplement: Supplementary file 2 — Supplementary file2 (DOCX 15 kb) [file 426_2021_1541_MOESM2_ESM.docx]

***Appendix***

*Figure A1. Survey Prompt (Example, referring to Map A).*

On your left, you see a map of the city. The city is subdivided into an internal and an external part, separated by a city wall. The creek Dinkel flows through the city from the South to the Northwest.

There is a subway in the city. Altogether, there are four subway lines, which are shown on the map by different colors. The yellow line extends from the Southwest to the North, and the red line from the Southwest to the Southeast. The blue line extends from the South to the East and the green line from the East to the Northeast.

In the center of the city, there is the police office and the Saint Peter Church. North from there is the Congress Hall. In the Northwest, you see a shopping mall. In the Western part of the city, you see a small forest and in the Southeast, the municipal museum.

*Figure A2. Connect Prompt* *(Example, referring to Map A).*

On your left, you see a map of the city. At lower left, you see the station Friedberg, top left Marienhöhe. In order to get from Friedberg to Marienhöhe, you have to go to Bastei via Südturm with the red line, change there to the blue line and go to Schloss, change there to the yellow line and go via Universität to Marienhöhe. You see altogether 6 stations, change 2 times, and use 3 lines.

On the right in the middle you find Niederfeld and Theaterplatz. In order to get from Niederfeld to Theaterplatz, you have to go to Ostbahnhof via Südring and Markt with the green line and change at Ostbahnhof to the blue line. You see altogether 5 stations, change 1 time, and use 2 lines.
